# Efficacy and Clinical Outcomes of Crizotinib in Patients with ROS1-Rearranged NSCLC: A Multicenter Study

**DOI:** 10.3390/medicina61030490

**Published:** 2025-03-12

**Authors:** Alper Topal, Goncagul Akdag, Sedat Yildirim, Oguzcan Kinikoglu, Deniz Isik, Gizem Yildirim, Salih Tunbekici, Fatih Kus, Aydın Acarbay, Murad Guliyev, Nargiz Majidova, Yasin Kutlu, Mustafa Erman, Hatice Odabas, Nedim Turan, Nuri Karadurmus

**Affiliations:** 1 Department of Medical Oncology, Health Science University, Gulhane Research and Training Hospital, Ankara 06010, Turkey; gizemyildirim_@hotmail.com (G.Y.); drnkaradurmus@yahoo.com (N.K.); 2 Department of Medical Oncology, Kartal Dr. Lütfi Kirdar City Hospital, Health Science University, Istanbul 34865, Turkey; akdaggoncagul@gmail.com (G.A.); rezansedat@hotmail.com (S.Y.); ogokinikoglu@yahoo.com (O.K.); dnz.1984@yahoo.com (D.I.); odabashatice@yahoo.com (H.O.); turan.nedim@hotmail.com (N.T.); 3 Division of Medical Oncology, Departmant of Internal Medicine, Ege Universitiy, Izmir 35100, Turkey; slhtnbkc@yahoo.com; 4 Department of Medical Oncology, Hacettepe University Oncology Institue, Ankara 06230, Turkey; fatihkush@hotmail.com (F.K.); ermanm1968@gmail.com (M.E.); 5 Division of Medical Oncology, Departmant of Internal Medicine, Istanbul Medeniyet University, Istanbul 34730, Turkey; aydinacarbay@gmail.com; 6 Division of Medical Oncology, Departmant of Internal Medicine, Istanbul University-Cerrahpasa, Istanbul 34098, Turkey; drguliyev892@gmail.com; 7 Department of Medical Oncology, Marmara University Pendik Training and Research Hospital, Istanbul 34854, Turkey; nergiz.mecidova1991@gmail.com; 8 Department of Medical Oncology, Tokat State Hospital, Tokat 60000, Turkey; dryasinkutlu@gmail.com

**Keywords:** ROS1, lung cancer, crizotinib

## Abstract

*Background and Objectives:* ROS1 rearrangement is a rare but targetable alteration in non-small-cell lung cancer (NSCLC), occurring in 1–2% of cases. Crizotinib, a tyrosine kinase inhibitor, has demonstrated efficacy in clinical trials, but real-world data remain limited. This study evaluates the safety and efficacy of crizotinib in ROS1-rearranged NSCLC patients in a real-world setting. *Materials and Methods:* This multicenter, retrospective research included 43 individuals with advanced/metastatic NSCLC and confirmed ROS1 rearrangements. Patients were treated with crizotinib in first- or second-line settings. Efficacy endpoints included progression-free survival (PFS), overall survival (OS), objective response rate (ORR), and disease control rate (DCR). Safety was assessed using Common Terminology Criteria for Adverse Events (CTCAE) version 5.0. *Results:* The median follow-up was 45.8 months. The ORR for first-line crizotinib was 72.1%, with a DCR of 79%. The median PFS was 20.9 months (95% CI: 6.02–35.69), and the median OS was 52.7 months (95% CI: 13.08–92.31). ECOG performance status was a significant prognostic factor for ORR (*p* = 0.02). The most common adverse events were fatigue (16.2%), elevated transaminases (13.9%), and vision disorders (11.6%). All reported adverse events were grade 1 or 2, with no grade ≥ 3 events observed. *Conclusions:*Crizotinib demonstrated significant efficacy and a favorable safety profile in real-world individuals with ROS1-rearranged NSCLC. These findings align with pivotal trials, underscoring crizotinib’s role as a standard treatment for this molecular subset. Further prospective studies are warranted to explore intracranial efficacy and long-term outcomes.

## 1. Introduction

About 85% of all occurrences of lung cancer are non-small-cell lung cancer (NSCLC), making it one of the most prevalent and significant causes of mortality globally [1]. The identification of driver mutations in non-small-cell lung cancer has completely transformed and altered treatment algorithms. Approximately 60% of lung cancer patients in Western societies and 80% of patients in Asian populations now have targetable molecular changes [2]. A tiny percentage of individuals with non-small-cell lung cancer (NSCLC) have one of these mutations, C-ros oncogene 1 (ROS1). Its potential as a therapeutic target in metastatic illness has garnered a lot of attention. About 1% to 2% of NSCLC patients had ROS1 fusion mutations [3,4]. ROS1 and anaplastic lymphoma kinase (ALK) have similar patient demographics. It is mostly detected in adenocarcinoma and is more common in Asian patients, younger patients, female patients, and non-smokers [2]. Being a true oncogenic driver, ROS1 often excludes other major driver changes [5].

The oral tyrosine kinase inhibitor (TKI) crizotinib targets ROS1 tyrosine kinase, mesenchymal–epithelial transition factor receptor (MET), and ALK (TKI) [6,7]. The binding regions of ALK and ROS1, to which crizotinib attaches, share similar three-dimensional structures. Because both tyrosine kinases have structural similarities, the same small drug can inhibit both ALK and ROS1 [8]. The first targeted treatment for non-small-cell lung cancer (NSCLC) with ROS1 rearrangements, crizotinib, was authorized by the US Food and Drug Administration in March 2016, marking a significant milestone in treating this condition. Crizotinib has demonstrated high response rates and prolonged survival outcomes. The normal dose of 250 mg given twice daily produced a median progression-free survival (PFS) of 19.2 months and an objective response rate (ORR) of 72% in the phase I PROFILE 1001 study [9]. Likewise, a phase II study in an East Asian population found a median overall survival (OS) of 33 months, a median PFS of 16 months, and an ORR of 72% [10].

Despite the encouraging outcomes of randomized clinical trials, there remain obstacles in using these data in actual clinical settings. There may be differences between trial efficacy and real-world effectiveness because patients in everyday practice frequently have a variety of comorbidities and traits that are under-represented in carefully controlled research settings [11,12]. Early real-world studies have reported consistent outcomes, with ORR ranging from 65% to 87% and median PFS spanning 9 to 23 months, highlighting the potential efficacy of the therapy in routine clinical practice [13,14,15,16].

We aimed to share the results of our multicenter study with the world by evaluating the safety and efficacy profile of crizotinib in individuals having NSCLC with ROS1 rearrangement. By synthesizing the latest evidence, we aim to provide valuable information for ROS1 rearrangement-positive patients having metastatic NSCLC.

## 2. Materials and Methods

### 2.1. Patients

This research is a multicenter, retrospective study. Individuals aged 18 years and older with advanced/metastatic NSCLC with confirmed ROS1 rearrangement (fluorescence in situ hybridization (FISH) and next-generation sequencing (NGS)) who received crizotinib treatment were included in our study. Individuals lacking full medical records or those with secondary cancers were not included. Demographics of the participants, including age, treatments received, pathological subtype, performance status, and adverse events, were obtained from medical records.

### 2.2. Study Endpoints, Response Criteria, and Safety Evaluation

The PFS, OS, ORR, duration of response (DOR), and disease control rate (DCR) were calculated in order to evaluate efficacy. PFS is used to define the period of time between the start of crizotinib therapy and either the first progression of the illness or death. At their last follow-up, patients who were still alive and not progressing at the time of analysis were censored. The time interval between the initial crizotinib dosage and the date of radiological progression, death, or the last documented visit was known as the PFS. DOR was used to define the interval from commencement (when either CR or PR is first determined) to progression or death, whichever happens first. OS was the period from the first crizotinib dosage to the date of death from any cause or the final visit. ORR was defined as the proportion of patients with CRs and PRs, whereas DCR was defined as the proportion of patients with a complete response (CR), partial response (PR), and stable disease (SD). The initial tumor response assessment was conducted three months after the initiation of crizotinib treatment, with subsequent evaluations performed at three-months intervals using the Response Evaluation Criteria in Solid Tumors (RECIST, version 1.1) [17].

Patient records provided the source of adverse events, which were then assessed and grouped using the Common Terminology Criteria for Adverse Events, version 5.0 [18].

### 2.3. Statistical Analysis

For statistical analyses, IBM SPSS Statistics for Windows, Version 25.0 (IBM Corp., Armonk, NY, USA), was employed. We used descriptive statistics to assess the frequency distributions of the gathered information. Frequencies and percentages were used to represent categorical data, and median values with ranges (minimum–maximum) were employed for continuous variables. The computation of overall and progression-free survival was performed using the Kaplan–Meier approach. A two-sided *p*-value of less than 0.05 was taken for statistical significance, and a 95% confidence interval (CI) was selected.

### 2.4. Ethical Approval

Every technique employed in this investigation involving human participants complied with the Helsinki Declaration of 1964, its subsequent revisions, similar ethical standards, and the ethical requirements of the institutional and national research committees. The Ethics/Institutional Review Board of the Kartal Dr. Lütfi Kirdar City Hospital in Istanbul, Türkiye, gave its approval to the study. The Approval Number is 2024/010.99/9/17, 25 October 2024.

## 3. Results

### 3.1. Patients Characteristics

The study included 43 patients, 28 (65.1%) males and 15 (34.9%) females. The median age of the patients was 51 years, with a normal distribution. Twenty-one patients (48.8%) were never smokers, while 22 (51.2%) were former or current smokers. Among smokers, the median pack-year history was 25 (2–50 pack-years). Forty-one patients (95.3%) had adenocarcinoma histology, while two (4.7%) had squamous histology. The most common tumor location was the right lower lobe, observed in 14 patients (32.7%). At diagnosis, three patients (7%) were in stage I-II, five patients (11.6%) were in stage III, and 35 patients (81.4%) had de novo metastatic disease. The most frequent site of metastasis was the brain, observed in 15 patients (34.9%), followed by bone metastases in 18 patients (41.9%) (characteristics of patients are given in Table 1).

An analysis of comorbidities revealed that 13 patients had hypertension, 5 patients had chronic obstructive pulmonary disease, 4 patients had ischemic heart disease, and 3 patients had diabetes mellitus.

Regarding initial treatments, 35 patients (81.3%) received first-line crizotinib therapy, while 8 (18.7%) were treated with first-line chemotherapy. Of the 43 participants, 22 (51.1%) were able to receive second-line treatment. Among the patients who experienced progression on crizotinib, 10 (23.2%) received lorlatinib, and 4 (9.3%) received chemotherapy. All eight patients (18.7%) who progressed after first-line chemotherapy were treated with crizotinib in the second line. Six patients (13.9%) proceeded to third-line treatment, of whom four (9.3%) received lorlatinib, and two (4.6%) received chemotherapy (Table 2).

### 3.2. Efficacy

**Figure 1:**
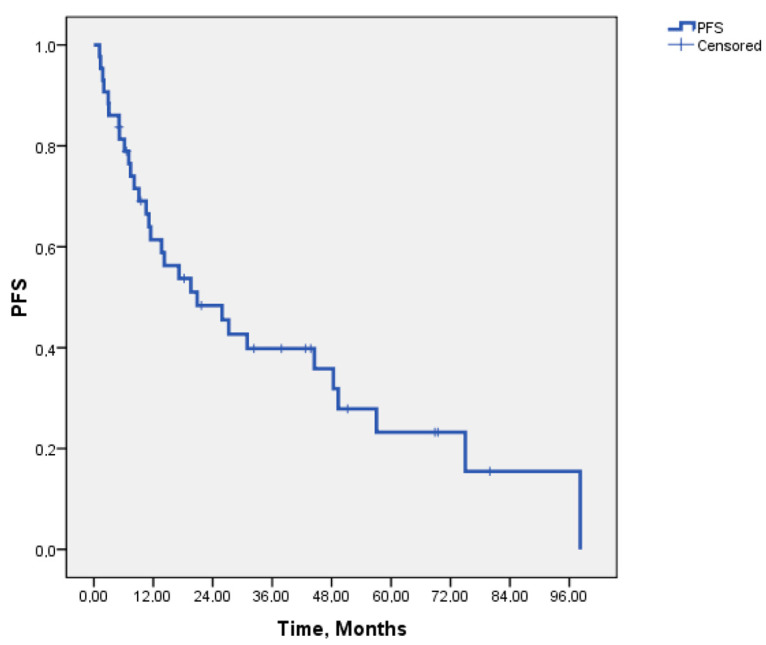
Kaplan–Meier curves of the progression-free survival of the 43 ROS1-rearranged NSCLC patients treated with crizotinib.

**Figure 2:**
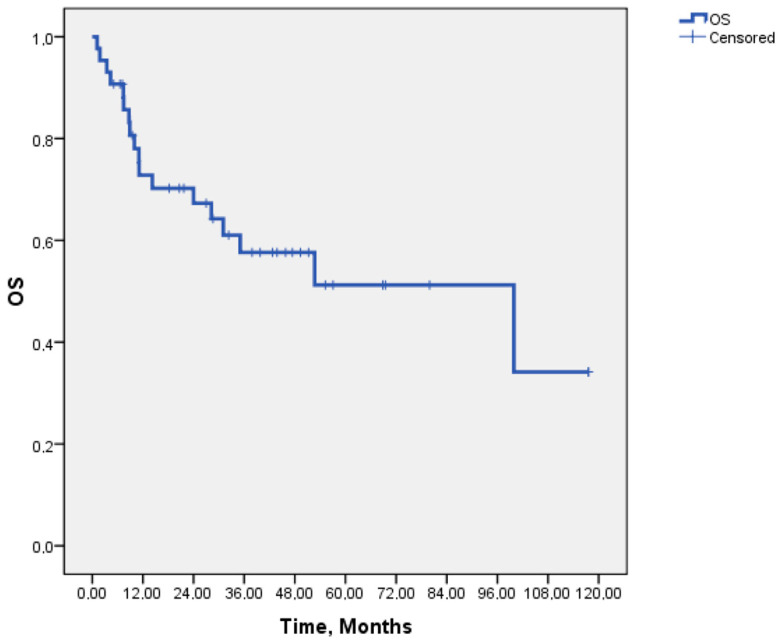
Kaplan–Meier curves of the overall survival of the 43 ROS1-rearranged NSCLC patients treated with crizotinib.

The median follow-up duration of our study was 45.8 months. The median PFS for patients receiving crizotinib was 20.9 months (95% CI: 6.02–35.69), while the median DOR was 20.4 months (95% CI: 14.6–26.1). The median OS was 52.7 months (95% CI: 13.08–92.31), with OS rates of 90.5% at 6 months, 72% at 12 months, 66% at 24 months, and 56.4% at 48 months. Kaplan–Meier survival curves for PFS and OS are presented in Figure 1 and Figure 2.

In the analysis of the two patients with a squamous cell carcinoma, one patient was 78 years old with de novo metastatic disease and received crizotinib treatment. He died after 1.8 months while receiving crizotinib treatment. The other patient was 52 years old and achieved PFS for 1.35 months with first-line crizotinib and is still under follow-up with third-line lorlatinib treatment.

**Figure 3:**
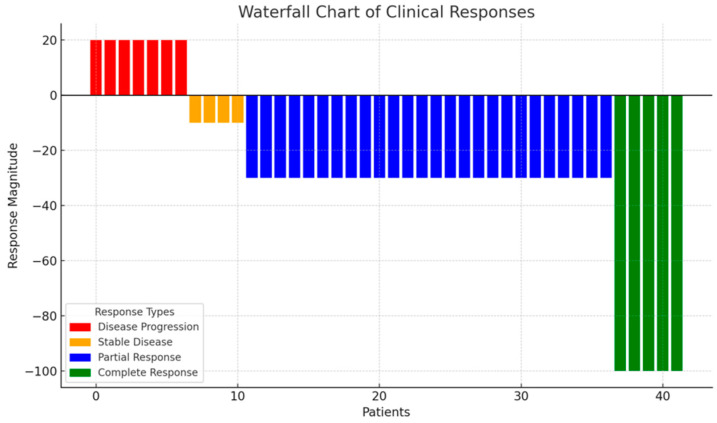
Waterfall chart of clinical response.

The ORR for first-line crizotinib therapy was 72.1%, with a DCR of 79%. Five patients (11.6%) achieved CR, 26 patients (60.5%) PR, and four patients (9.3%) SD, while eight patients (18.6%) experienced disease progression (Table 3). The ORR for second-line crizotinib therapy was 62.5%. Patient responses to crizotinib treatment are illustrated in a waterfall plot in Figure 3.

When factors influencing the ORR were analyzed (Table 4), it was found that gender, age, smoking history, baseline disease status, presence of brain metastases at diagnosis, the treatment line at which crizotinib was initiated, and tumor location did not significantly impact ORR. However, a statistically significant association was observed between ECOG PS and ORR (*p* = 0.02). Multivariate analysis revealed that ECOG PS was an independent prognostic factor influencing ORR (OR: 0.15, 95% CI: 0.02–0.78, *p* = 0.02). None of the other evaluated factors demonstrated a significant effect on ORR.

### 3.3. Safety

Fatigue was the most frequent adverse effect, occurring in 16.2% of participants. Elevated transaminases were the second most frequent adverse event., occurring in 13.9% of patients. Vision disorders were reported in 5 participants (11.6%), nausea in 4 patients (9.3%), serum creatinine elevation in 2 patients (4.3%), anemia in 4 patients (9.3%), diarrhea in 2 patients (4.6%), and edema in 1 patient (2.3%). Treatment was temporarily interrupted in 5 patients (4.3%) due to adverse events but was subsequently resumed (Table 5).

## 4. Discussion

Since its approval in 2016, crizotinib has been used as a first-line treatment for individuals with advanced NSCLC having ROS1 rearrangement. This multicenter, retrospective analysis provides actual data on the clinical characteristics and results of 43 participants having NSCLC who were treated with crizotinib and had ROS1 rearrangement.

Crizotinib showed a 72% ORR, a 19.2 months median PFS, and a 51.4 months median OS in the pivotal PROFILE 1001 trial [9]. Similarly, our study yielded comparable results, supporting the findings of PROFILE 1001. In our study, the ORR was 72%, the PFS median was 20.9 months, and the OS median was 52.7 months. Consistent ORRs have also been reported in other studies, such as a large phase II trial involving 127 East Asian patients and a study conducted in South Korea [3,10]. In contrast, the phase II AcSe study conducted in France, which included 37 patients, reported an ORR of 68% and a PFS median of 5.5 months [14], including 25% of participants with an ECOG PS of two, which might explain these results. Studies assessing crizotinib’s effectiveness in individuals with NSCLC having ROS1 rearrangements are summarized in Table 6. Our findings also support the impact of ECOG PS on treatment outcomes. In our study, participants with ECOG PS 0-1 had an ORR of 80%, while those with ECOG PS 2 had an ORR of 37.5%. The median DOR in the updated analysis of PROFILE 1001 was 24.7 months [9], while it was 19.7 months in the East Asian study involving 127 patients [3]. In this present research, the median DOR was 20.4 months. In the PROFILE 1001 trial, the median age of the participants was 55 years [9]. In contrast, the median age of our patient cohort was 51 years, consisting of a relatively younger population. Additionally, 57% of the patients in PROFILE 1001 were female, and 70% were non-smokers [9]. In this present research, only 35% of the patients were female, and 49% were non-smokers, which was relatively lower.

In our real-world study data, the rate of brain metastases was 34.9%, notably higher than that reported in most other studies (3.2–23.1%) [10,13,15,16]. This may have influenced our outcomes. Hong et al. demonstrated that continuing crizotinib treatment while administering local therapies to the brain in patients with brain progression could contribute to disease control [22]. Similarly, in our study, seven patients (16%) who experienced brain progression while on crizotinib received local therapies and continued crizotinib treatment. However, among these patients, only two achieved prolonged OS, of 46 and 57 months, respectively.

In our study, the safety profile of crizotinib was better compared to previous studies involving ROS1-positive and ALK-positive lung cancer individuals [3,9,10,23,24,25]. It is possible to attribute this to the retrospective approach in our research. Similarly, a comparable toxicity profile was observed in the series by Joshi et al., which included 22 patients [26]. All reported adverse events (AEs) in our study were grade 1 or 2 in severity, with no grade 3, 4, or 5 treatment-related adverse events (TRAEs) observed. This underscores the tolerability of crizotinib. The adverse events associated with crizotinib were controllable with brief dosage decreases or temporary treatment interruptions.

In the PROFILE 1001 trial, the adverse events that were most often documented were vision disorders (87%) and elevated transaminases (36%) [9]. In contrast, in our study, fatigue (16.2%) and elevated transaminases (13.9%) were the most common adverse effects. Vision disorders were only observed in five patients (11.6%). Our investigation found no new safety signals for crizotinib.

## 5. Conclusions

Our multicenter, retrospective study is one of the few real-world data reports demonstrating crizotinib’s safety and efficacy profile in NSCLC individuals having ROS1 rearrangements. Because of the rarity of ROS1 rearrangements, our study, which includes 43 patients, holds significant value. Compared to other studies in the literature, our sample size is larger than that of many previous reports [13,14,19,25,27]. Consistent with earlier studies, most patients in our cohort achieved durable responses to crizotinib treatment, regardless of their demographic or baseline clinical characteristics. It was demonstrated that crizotinib worked efficiently in both first-line and second-line situations. Overall, our study corroborates the findings of the PROFILE 1001 trial. Moreover, it is among the rare studies that include OS data.

In conclusion, ROS1 rearrangement defines a distinct molecular subgroup of NSCLC in which crizotinib is highly effective. Crizotinib has led to durable clinical responses in most patients, and has been associated with grade 2 or lower adverse events. Our study demonstrates that crizotinib is an effective and safe treatment option for advanced/metastatic NSCLC patients with ROS1 rearrangements.

### Limitations

There are numerous restrictions to our investigation. Firstly, it is retrospective, which may introduce inherent biases. Secondly, we were unable to assess intracranial efficacy. Finally, our findings may not be as broadly applicable as they may be due to our comparatively limited sample size.

## Figures and Tables

**Table 1:** Baseline characteristics of patients with ROS1-rearranged non-small-cell lung cancer.

Baseline Characteristics	n = 43 (%)
Median age (range) [years]	51 (32–79)
Age	
<65	34 (65.1)
≥65	9 (20.9)
Sex	
Male	28 (65.1)
Female	15 (34.9)
ECOG PS	
0	21 (48.8)
1	14 (32.6)
2	8 (18.6)
Smoking history	
Never	21 (48.8)
Former or current	22 (51.2)
Smoking packet/years, median (min–max)	25 (2–50)
Comorbidity	
Absence	25 (58.1)
Presence	18 (41.9)
Histological type at initial diagnosis	
Adenocarcinoma	41 (95.3)
Squamous cell carcinoma	2 (4.7)
Localization	
Right upper lobe	5 (11.6)
Right middle lobe	5 (11.6)
Right lower lobe	14 (32.7)
Left upper lobe	9 (20.9)
Left lower lobe	10 (23.2)
Stage at diagnosis	
Stage I–II	3 (7)
Stage III	5 (11.6)
Stage IV	35 (81.4)
Primary Lesion PET/CT SuvMax	
Median (min–max)	11.5 (4–23)
Disease status	
Denovo metastatic	35(81.4)
Recurrent metastatic	8 (18.6)
Metastatic site	
Brain	15 (34.9)
Contralateral lung	9 (20.9)
Liver	8 (18.6)
Bone	18 (41.9)
Adrenal glands	7 (16.3)

ECOG PS: eastern cooperative oncology group performance status, PET/CT: positron emission tomography/computed tomography.

**Table 2:** Initial and second-line therapies in patients with ROS1-rearranged non-small-cell lung cancer.

Initial Therapy	n = 43 (%)	Second-Line Therapy (n = 22)	Third-Line Therapy (n = 6)
Crizotinib	35 (81.3)	Lorlatinib (10), Chemotherapy (4)	Lorlatinib (1), Chemotherapy (1)
Cytotoxic chemotherapy	8 (18.7)	Crizotinib (8)	Lorlatinib (3), Chemotherapy (1)

**Table 3:** Summary of efficacy endpoints and survival probability.

Endpoints	ROS1-Rearranged NSCLC (n = 43)
ORR, %	72.1
DCR, %	79.0
CR, n (%)	5 (11.6)
PR, n (%)	26 (60.5)
SD, n (%)	4 (9.3)
PD, n (%)	8 (18.6)
Median DOR, months (95% CI)	20.4 (14.6–26.1)
Median PFS, months (95% CI)	20.9 (6.02–35.69)
Median OS, months (95% CI)	52.7 (13.08–92.31)
Deaths, n (%)	18 (58.1)
Survival probability, %	
6 months	90.5
12 months	72.0
24 months	66.0
48 months	56.4

ROS1: C-ros oncogene 1, NSCLC: non-small-cell lung cancer, ORR: objective response rate, DCR: disease control rate, CR: complete response, PR: partial response, SD: stable disease, PD: progressive disease, DOR: duration of response, PFS: progression-free survival, OS: overall survival, CI: confidence interval.

**Table 4:** Clinicopathological characteristics with respect to objective response rate achievement.

	Total Crizotinib (n = 43)		
Characteristic	No of Patients	ORR, %	*p*
Sex			
Male	19 of 28	67.8	0.49
Female	12 of 15	80.0	
Age group			
<65	26 of 34	76.5	0.23
≥65	5 of 9	55.5	
Smoking history			
No	15 of 21	71.4	0.99
Yes	16 of 22	72.7	
ECOG PS			
0–1	28 of 35	80.0	0.02
2	3 of 8	37.5	
Disease status			
Denovo metastatic	25 of 35	71.4	0.9
Recurrent metastatic	6 of 8	75.0	
Brain metastases at baseline			
Absence	11 of 15	73.3	0.99
Presence	20 of 28	71.4	
TKI received a treatment line			
First-line	26 of 35	74.2	0.66
Second-line	5 of 8	62.5	
Tumor Localization			
Right lung	18 of 24	75.0	0.73
Left lung	13 of 19	68.4	

ORR: objective response rate, ECOG PS: eastern cooperative oncology group performance status, TKI: tyrosine kinase inhibitor.

**Table 5:** Adverse events with crizotinib.

	Number (%)
Adverse Event *	
Elevated transaminases	6 (13.9)
Vision disorder	5 (11.6)
Nausea	4 (9.3)
Elevated blood creatinine	2 (4.6)
Anemia	4 (9.3)
Diarrhea	2 (4.6)
Edema	1 (2.3)
Fatigue	7 (16.2)
Drug interruptions	
Interruption of treatment	5 (4.6)

* Adverse events were categorized, and graded as per the Common Terminology Criteria for Adverse Events, version 5.0.

**Table 6:** Summary of crizotinib studies in patients with ROS1-rearranged non-small-cell lung cancer.

	n	ORR %	mPFS (mo) (95% CI)	mOS (mo) (95% CI)	1-Year OS
PROFILE 1001 [9]	53	72	19 (15–39)	51 (29-NR)	-
EUROS-1 [13]	31	80	9	-	-
AcSe [14]	36	47	6 (4–9)	17 (9–33)	-
EUCROSS [19]	34	70	20 (8-NR)	NR	83%
METROS [15]	26	65	23 (15–30)	NR	-
East Asian [10]	127	72	16 (13–24)	33	83%
Shanghai [16]	30	87	18 (6–30)	NR	81%
Beijing [20]	56	84	15 (11–19)	NR	-
China [21]	168	86	18	-	-
Topal et al.	43	72	21 (6–35)	53 (13–92)	72%

ORR: objective response rate, mPFS: median progression-free survival, mOS: median overall survival, CI: confidence interval.

## Data Availability

The data presented in this study are available on request from the corresponding author.
